# Bayesian Clinical Trial Design for Distributional Outcomes with Noncompliance

**DOI:** 10.3390/e28091040

**Published:** 2026-09-21

**Authors:** Xin Guo, Zhi Yang, Siyuan Li, Weining Shen

**Affiliations:** 1 School of Statistics and Data Science, Shanghai University of Finance and Economics, Shanghai 200433, China; 2 Santa Margarita Catholic High School, Irvine, CA 92688, USA; 3 Department of Statistics, Donald Bren School of Information and Computer Sciences, University of California, Irvine, CA 92697, USA

**Keywords:** Bayesian design, Wasserstein distance, causal effect, noncompliance, principal stratification

## Abstract

We consider sequential monitoring in early-phase clinical trial designs with patient noncompliance. Unlike most existing methods that focus on modeling a single endpoint, we consider the entire outcome distribution and propose a Bayesian causal effect estimation method based on the Wasserstein distance. Using a principal stratification framework and random forest to classify patient compliance statuses, we quantify a distributional causal-effect magnitude among Compliers by calculating the Wasserstein distance between the barycenters of their potential outcome distributions. The proposed method also adaptively determines early trial termination based on accumulated outcomes. Simulation results show favorable operating characteristics under the scenarios investigated, with the proposed analysis remaining close to the oracle benchmark as noncompliance increases.

## 1. Introduction

One of the primary objectives of early-phase clinical trials is to evaluate the safety and preliminary efficacy of new drugs. This allows researchers to determine whether to invest in and advance larger-scale development. Interim monitoring is standard in clinical trials to evaluate therapeutic utility. Early termination occurs when a treatment demonstrates ineffectiveness or harm, thereby protecting participants and conserving resources. Two-stage and multi-stage designs have played a central role in the planning of early-phase clinical trials. Pioneering works include the two-stage design by Gehan [1] and Fleming’s framework [2]. Simon [3] significantly advanced this line of work by proposing the optimal two-stage design that minimizes the expected sample size. Building on these foundational designs, later works have proposed multi-center trial frameworks [4], extensions to three-stage designs [5], and adaptive trial strategies [6]. Bayesian methods have provided flexible alternatives for interim monitoring; for example, Thall and Simon [7] advocated Bayesian continuous monitoring using posterior probability thresholds to enable early stopping based on estimated treatment effects.

In recent years, there has been growing interest in addressing the critical challenge of noncompliance in early phase trial designs based on causal inference frameworks. Shen et al. [8] proposed a Bayesian sequential monitoring design based on causal effect estimation that allows for robust inference in two-arm randomized trials with noncompliance. Ren et al. [9] further employed Bayesian Additive Regression Trees (BART) under the principal stratification framework to predict the patients’ compliance status and then use that for estimating average causal effects. Together, these studies show how explicitly modeling compliance and causal estimands can improve interim monitoring; however, they focus on scalar endpoints rather than on causal contrasts between distribution-valued outcomes.

The distinction between intention-to-treat (ITT) and principal stratification analyses is important in this setting: ITT preserves the randomized comparison and estimates the effect of treatment assignment, but does not generally identify the effect of treatment receipt among compliers when the received treatment differs from the assigned treatment; principal stratification instead targets causal effects within latent compliance groups, but at the cost of additional identifying assumptions such as monotonicity and exclusion. Our design adopts the latter framework, as the stopping decision is intended to reflect the treatment effect among patients who would comply with their assignment.

In this paper, we focus on trial designs with distributional outcomes, where the interest is to model the entire distribution of the outcomes rather than a single endpoint. Chronic disease trials illustrate the need for such approaches: in Schiaffini et al.’s telemedicine study [10], blood glucose outcome showed a non-monotonic trajectory over five years, yet analysis using traditional methods would ignore these fluctuations. Similarly, the study outcomes in a weight loss trial [11] also exhibit a nonlinear longitudinal pattern that cannot be captured by studying a single endpoint. Existing methodologies have focused mainly on univariate endpoints, neglecting the dynamic information inherent in longitudinal data.

Recent Bayesian phase II designs have begun to use longitudinal endpoints directly, for example by modeling subject-specific trajectories and monitoring a scalar trajectory summary [12]. While these approaches demonstrate the value of borrowing information across repeated observations, their decision criteria are usually based on a mean trajectory or an area-under-the-curve summary. Our focus is different; in this paper, we define a causal contrast for distribution-valued outcomes and incorporate noncompliance through principal stratification.

Our aim is to extend the early-phase clinical trial design framework to distribution-valued outcomes using a causal estimand defined through Wasserstein barycenters. In the simulation studies, we evaluate the average sample size and early stopping proportion under different outcome trajectories and compliance rates. Under the examined scenarios, the proposed principal stratification analysis follows the oracle Gold benchmark more closely than the ITT analysis as compliance decreases; however, ITT targets the effect of randomized assignment, whereas the proposed analysis targets a distributional CACE magnitude among compliers. Therefore, their operating characteristics reflect different estimands and should not be interpreted as evidence that one approach is intrinsically superior. Comparisons with a mean-only summary further suggest that the Wasserstein summary can retain trajectory information that may be attenuated by averaging.

The remainder of this paper is organized as follows: Section 3 introduces the proposed design under compliance considerations and details the associated stopping rules; Section 4 evaluates the performance of our method through extensive simulation studies; Section 5 conducts sensitivity analyses to assess robustness under varying assumptions; finally, Section 6 concludes with a discussion of potential limitations and outlines directions for future research.

## 2. A Motivating Example

Obesity is characterized by excess body weight and adiposity, typically assessed using the body mass index (BMI), with values of 30 or above classified as obese. According to the World Health Organization, obesity has become one of the most critical global public health concerns, with significant implications for morbidity, mortality, and quality of life. In 2020, more than 2.2 billion adults, representing 42.3% of the world’s adult population, were overweight or obese, while 22.4% of children and adolescents were similarly affected. In addition to being a prevalent health problem, obesity represents a prototypical chronic disease in which the clinical course is marked by sustained physiological and behavioral changes. Empirical evidence from interventions in obese adults, including intermittent fasting, indicates that weight trajectories often exhibit complex longitudinal patterns such as initial decreases followed by partial regain and subsequent decline [13]. As a result, the evaluation of treatment efficacy should not be confined to outcomes measured at a single time point, e.g., mean weight change at the end of follow-up, but should instead be based on the entire outcome distributions.

**Figure 1:**
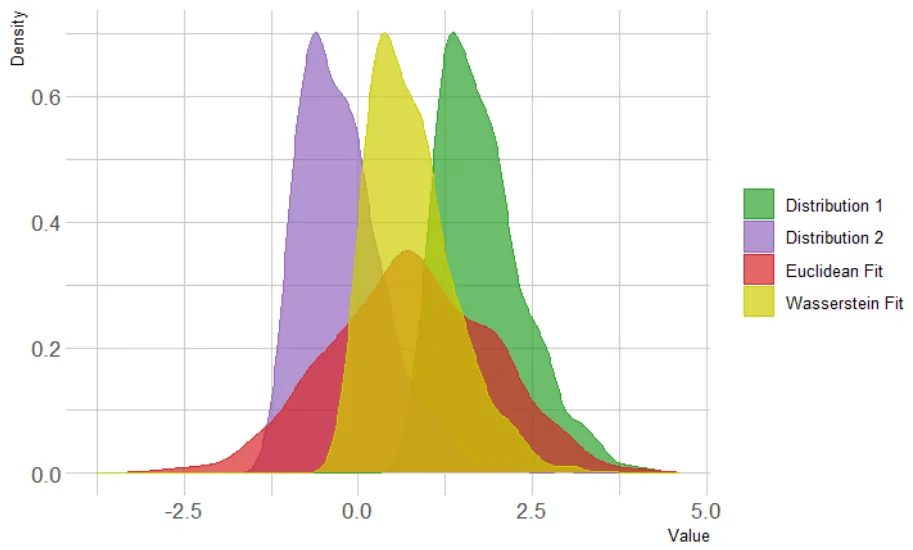
Comparison between the Wasserstein barycenter and Euclidean mean of two distribution functions.

Here, we consider an illustrative example motivated by an intermittent-fasting trial for adults with obesity [14]. Figure 1 presents two outcome distributions with similar shapes but different locations. In this illustration, the Euclidean average smooths some of the distributional structure, whereas the Wasserstein barycenter retains the location and shape changes more clearly. This example motivates the use of a distributional summary when clinically relevant longitudinal features may be attenuated by a mean-only analysis.

## 3. Method

### 3.1. Observed Data, Estimand, and Rationale

For subject i=1,…,n, let $X_{i}$ denote baseline covariates, $Z_{i}\in \left\{0,1\right\}$ the randomized treatment assignment, $D_{i}$ the treatment actually received, and

$$Y_{i}={\left\{Y_{i}\left(t_{1}\right),\ldots ,Y_{i}\left(t_{p}\right)\right\}}^{T}\in R^{p}$$

the longitudinal outcome vector observed at *p* time points. The observed data are

$$D_{n}=\left\{\left(Y_{i},X_{i},Z_{i},D_{i}\right):i=1,\ldots ,n\right\}.$$

The objective is to monitor a distributional treatment effect magnitude among compliers while accounting for posterior uncertainty in the outcome model. The random forest stratum classifications are treated as fixed inputs in the current implementation.

A distributional summary is useful when two groups have similar scalar means but differ in spread or shape. For distributions on the real line, the 2-Wasserstein distance has the quantile representation

$$W_{2}^{2}\left(F_{1},F_{2}\right)=\int_{0}^{1}{\left\{F_{1}^{-1}\left(u\right)-F_{2}^{-1}\left(u\right)\right\}}^{2}\;du.$$

This representation makes the barycenter computationally tractable and the corresponding causal map interpretable as a quantile-by-quantile displacement. In the degenerate case in which every distribution is a point mass, the Wasserstein barycenter reduces to the ordinary mean and the effect magnitude reduces to the absolute classical mean contrast. Figure 1 illustrates why the Wasserstein barycenter can retain distributional structure that is lost under a pointwise Euclidean average.

### 3.2. Defining Causal Effects for Distribution Functions

We start with a review of the Wasserstein barycenter and a discussion of how to define causal effects for distribution functions, following [15]. The Wasserstein distance, also known as the Earth Mover’s Distance, is a measure of the difference between two probability distributions. Let *X* and *Y* be random variables in an interval I of R, and let $\lambda_{1}$ and $\lambda_{2}$ be their distribution functions. The 2-Wasserstein distance between $\lambda_{1}$ and $\lambda_{2}$ is defined as

$$W_{2}\left(\lambda_{1},\lambda_{2}\right)={\left(\underset{\lambda_{12}\in \Lambda \left(\lambda_{1},\lambda_{2}\right)}{\inf}\int_{I\times I}{\left(x-y\right)}^{2}d\lambda_{12}\left(x,y\right)\right)}^{1/2},$$

where $\Lambda (\lambda_{1},\lambda_{2})$ denotes all possible joint distributions $\lambda_{12}$ for which the marginal distributions are $\lambda_{1}$ and $\lambda_{2}$, respectively. Based on this, the Wasserstein space of order 2 on I is defined as the space of distribution functions on I with finite second moments

$$W_{2}\left(I\right)=\left\{\lambda \;\mathrm{is}\;a\;\mathrm{distribution}\;\mathrm{function}\;\mathrm{on}\;I:\int_{I}t^{2}d\lambda \left(t\right)<\infty \right\}$$

endowed with the 2-Wasserstein distance. Next, we introduce the definition of the Wasserstein barycenter. This refers to the “average” distribution of multiple probability distributions in the Wasserstein space. Given a set of probability distributions $\lambda_{1},\lambda_{2},\ldots ,\lambda_{n}$, its Wasserstein barycenter $\hat{\mu }$ is a probability distribution that satisfies

$$\hat{\mu }=\underset{v\in W_{2}\left(I\right)}{\arg\min}\sum_{i=1}^{n}\rho_{i}W_{2}^{2}\left(\lambda_{i},v\right),$$

where *v* is a candidate distribution over which the minimization is performed, $\hat{\mu }$ is the minimizing Wasserstein barycenter, and $\rho_{i}$ is a non-negative weight satisfying $\sum_{i=1}^{n}\rho_{i}=1$.

Following the notation in Lin et al. [15], let *a* represent the binary treatment indicator that takes values in {0,1} and let $Y\left(a\right)$ represent the outcome based on *a*, where $Y\left(1\right)$ and $Y\left(0\right)$ denote the outcome for the treatment group and control group, respectively. The Wasserstein barycenter of $Y\left(1\right)$ and $Y\left(0\right)$ is defined as

(1) $${\hat{\mu }}_{a}=\underset{v\in W_{2}\left(I\right)}{\arg\min}\;E\left\{W_{2}^{2}\left(Y\left(a\right),v\right)\right\},\;a=0,1,$$

where Y(a) is a random distribution-valued outcome across subjects, so that ${\hat{\mu }}_{a}$ is the barycenter of a population of subject-level potential outcome distributions rather than the barycenter of one fixed distribution. Let ${\hat{\mu }}_{0}^{-1}$ and ${\hat{\mu }}_{1}^{-1}$ denote the quantile functions of the control and treatment Wasserstein barycenters, respectively. The causal effect map is defined as

$$\Delta^{\lambda }\left(\cdot \right)=\left({\hat{\mu }}_{1}^{-1}-{\hat{\mu }}_{0}^{-1}\right)\circ \lambda \left(\cdot \right),$$

where λ is a continuous reference distribution that places the two quantile functions on a common index. In particular, if λ is uniform on [0,1], then $\Delta^{\lambda }\left(u\right)$ is the treatment-minus-control difference at quantile level *u*. The map retains the direction and quantile-specific pattern of the contrast. Its scalar magnitude is the 2-Wasserstein distance between the two barycenters:

(2) $$ACE=W_{2}\left({\hat{\mu }}_{1},{\hat{\mu }}_{0}\right)=\left|\right|\Delta^{\lambda }{\left|\right|}_{\lambda }:={\left\{E_{U∼\lambda }{\left(\Delta^{\lambda }\right)}^{2}\left(U\right)\right\}}^{1/2}={\left\{\int {\left(\Delta^{\lambda }\right)}^{2}\left(u\right)d\lambda \left(u\right)\right\}}^{1/2}$$

where *U* has distribution function λ. By the probability integral transform, λ(U) is uniform on [0,1]; hence, the $L_{2}\left(\lambda \right)$ norm above equals the $L_{2}$ distance between the two barycenter quantile functions. We refer to this non-negative scalar as the distributional causal effect magnitude; within the complier stratum, it is the distributional CACE magnitude used for monitoring.

### 3.3. Compliance Settings

Now, consider a two-arm phase II trial. For subject *i*, let $X_{i}$ be the baseline covariates and $Z_{i}$ the assigned treatment. Here, $Z_{i}=0$ represents the control group and $Z_{i}=1$ represents the treatment group. Let $D_{i}\left(Z_{i}\right)$ be the actual treatment received by subject *i*. Then, $D_{i}\left(Z_{i}\right)=1$ indicates that the subject actually received treatment, while $D_{i}\left(Z_{i}\right)=0$ indicates that the subject did not receive treatment. Due to the presence of non-compliance, $D_{i}\left(Z_{i}\right)$ may not be equal to $Z_{i}$. Therefore, based on the principal stratification framework, subjects are classified into the following four categories based on their adherence status:

(1) Compliers: $D_{i}\left(0\right)=0$ and $D_{i}\left(1\right)=1$; this category of subjects complies with assigned experimental scheme.
(2) Always-Takers: $D_{i}\left(0\right)=1$ and $D_{i}\left(1\right)=1$; this category of subjects only complies with experimental group scheme.
(3) Never-Takers: $D_{i}\left(0\right)=0$ and $D_{i}\left(1\right)=0$; this category of subjects only complies with the control group scheme.
(4) Defiers: $D_{i}\left(0\right)=1$ and $D_{i}\left(1\right)=0$; this category of subjects does not comply with any assigned experimental scheme.

The monotonicity (no-defier) assumption requires $D_{i}\left(1\right)\geq D_{i}\left(0\right)$ for every subject. It rules out the stratum $D_{i}\left(0\right)=1,D_{i}\left(1\right)=0$, leaving $S_{i}=c$ (complier), $S_{i}=n$ (never-taker), and $S_{i}=a$ (always-taker). Monotonicity is an identifying assumption and is not implied by randomization. We additionally assume the following: (1) a stable unit treatment value assumption, meaning that there is no interference and no hidden version of either treatment; (2) randomized assignment, $Z_{i}⊥\left\{Y_{i}\left(0\right),Y_{i}\left(1\right),S_{i},X_{i}\right\}$; and (3) an exclusion restriction, under which assignment affects the outcome only through the treatment received.

Based on these assumptions, we define the distributional CACE magnitude using (2):

$$CACE=W_{2}\left(\hat{\mu }\left(S=c,Z=1\right),\hat{\mu }\left(S=c,Z=0\right)\right).$$

This is the Wasserstein distance between the barycenters of the potential outcome distributions for compliers under treatment and control. The random forest is used only as a working classifier of the latent principal stratum from baseline covariates, and is not itself the source of causal identification. Classification error may affect the stratum-specific outcome distributions; Section 5 examines one practically important form of compliance model misspecification.

### 3.4. Response Model and Posterior Sampling

We use a Gaussian process as a working model for the longitudinal outcome. Evaluating its mean function and covariance kernel K(·,·) at $t_{1},\ldots ,t_{p}$ gives a multivariate Normal model with

$$\Sigma_{pp}={\left\{K\left(t_{j},t_{k}\right)\right\}}_{j,k=1}^{p}.$$

Conditional on principal stratum $S_{i}=s$ and randomized arm $Z_{i}=z$, we write

$$Y_{i}|S_{i}=s,Z_{i}=z,\mu \left(s,z\right),\Sigma ∼N_{p}\left\{\mu \left(s,z\right),\Sigma \right\},$$

where $\mu \left(s,z\right)={\left\{\mu_{s,z}\left(t_{1}\right),\ldots ,\mu_{s,z}\left(t_{p}\right)\right\}}^{T}$ is the mean function evaluated at the observation times. An AR(1)-type working structure may be used for Σ to represent within-subject serial correlation.

Let $S_{i}^{\mathrm{RF}}$ be the working stratum classification produced by the random forest, $\phi_{p}\left(y;m,V\right)$ the *p*-variate Normal density, and S={c,n,a}. The observed-outcome likelihood contribution is then

(3) $$L_{i}=\sum_{s\in S}I\left(S_{i}^{\mathrm{RF}}=s\right)\;\phi_{p}\left\{Y_{i};\mu \left(s,Z_{i}\right),\Sigma \right\}.$$

Thus, for parameter collection θ={μ(s,z):s∈S,z∈{0,1}},

$$p\left(\theta ,\Sigma |D_{n},S^{\mathrm{RF}}\right)\propto p\left(\theta ,\Sigma \right)\prod_{i=1}^{n}L_{i}.$$

Equation (3) evaluates the Normal density at the observed $Y_{i}$. Integrating a normalized density over all of $R^{p}$ would equal one, and would not yield a data-dependent likelihood.

In the current implementation, the random forest is refitted at each interim analysis, after which the predicted labels $S_{i}^{\mathrm{RF}}$ are treated as fixed during posterior sampling of the outcome model. Consequently, uncertainty in principal-stratum classification is not fully propagated through the posterior distribution. A joint model for stratum membership and longitudinal outcomes or posterior averaging over possible stratum assignments would provide a more complete treatment of this uncertainty, and is left for future work.

For conjugate computation, we use

$$\mu \left(s,z\right)|\Sigma ∼N_{p}\left(m_{0},V_{0}\right),\;\Sigma ∼\mathrm{IW}\left(\nu_{0},S_{0}\right).$$

For the $n_{sz}$ subjects classified into stratum *s* and assigned to arm *z*, the conditional update for the mean is

$$\mu \left(s,z\right)|-∼N_{p}\left(m_{sz},V_{sz}\right),$$

where

$$V_{sz}={\left(V_{0}^{-1}+n_{sz}\Sigma^{-1}\right)}^{-1},\;m_{sz}=V_{sz}\left(V_{0}^{-1}m_{0}+n_{sz}\Sigma^{-1}{\bar{Y}}_{sz}\right).$$

The covariance update is

$$\Sigma |-∼\mathrm{IW}\left(\nu_{0}+n,\;S_{0}+\sum_{i=1}^{n}\left\{Y_{i}-\mu \left(S_{i}^{\mathrm{RF}},Z_{i}\right)\right\}{\left\{Y_{i}-\mu \left(S_{i}^{\mathrm{RF}},Z_{i}\right)\right\}}^{T}\right).$$

The Normal–inverse Wishart structure keeps the repeated interim updates computationally transparent [16]. Hyperparameters should be prespecified using clinically plausible outcome ranges or historical information; weakly informative choices can be used when reliable historical information is unavailable. Prior influence can be material in small samples. Standard posterior concentration under a correctly specified fixed-dimensional model does not imply more general robustness to covariance kernel or compliance model misspecification, and a broader prior sensitivity analysis remains an important direction for future work.

### 3.5. Stopping Rules

Based on the above settings, we consider a sequential trial framework in which subjects are enrolled in cohorts of five. Early stopping is implemented using the CACE magnitude described in Section 3.3. We prespecify a futility threshold *L* for the CACE magnitude and a posterior probability boundary *C*. The trial stops for futility if $P(CACE\leq L|D_{n})\geq C$. Both quantities should be determined using clinical knowledge and design calibration simulations.

With retained posterior draws *M* at interim sample size *n*, the posterior uncertainty is summarized by

$${\hat{p}}_{n}=\frac{1}{M}\sum_{m=1}^{M}I\left\{CACE^{\left(m\right)}\leq L\right\}.$$

**Figure 2:**
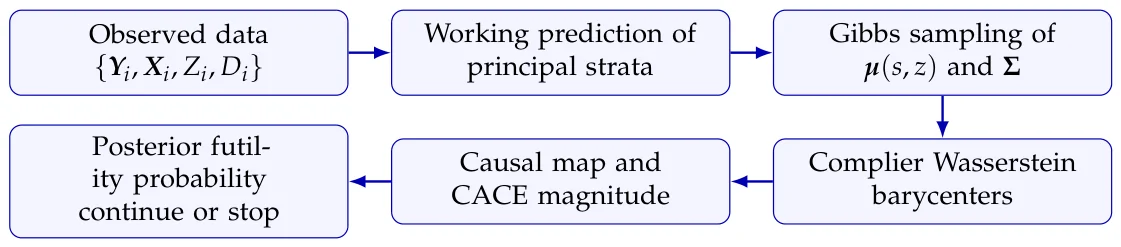
Workflow of the proposed sequential monitoring procedure. The random forest supplies a working principal-stratum classification; causal interpretation relies on the principal stratification assumptions rather than on the classifier alone.

The trial stops for futility when ${\hat{p}}_{n}\geq C$; otherwise, it continues to the next cohort, subject to the maximum sample size. Thus, the decision uses the complete posterior sample rather than only a point estimate. Pointwise posterior credible intervals for the CACE magnitude may be reported as descriptive summaries of posterior uncertainty. They are not used as the operating criterion of the design; the prespecified futility decision is based exclusively on whether ${\hat{p}}_{n}\geq C$. A complete workflow is provided in Figure 2.

Distribution-valued outcomes can be demanding in small samples. The present design mitigates but does not eliminate this difficulty by sharing information across repeated measurements through a hierarchical working model and by monitoring the scalar distance between two barycenters rather than estimating an unrestricted functional process. Its use requires sufficiently informative repeated measurements and a reasonable covariance model. The simulations begin monitoring at $n_{0}=30$, add cohorts of five, and cap enrollment at $n_{\max}=100$, thereby evaluating the intended early-phase sample size regime.

## 4. Simulation Studies

### 4.1. A Fasting Therapy Trial Example

We first describe a trial relevant to our study. The treatment is an intermittent fasting therapy for obese adults [14], with the primary goal of assessing the degree of weight loss and the effectiveness of an 8-h time-restricted eating regimen over 12 weeks compared to no intervention. The 8-h time-restricted eating condition requires participants to eat freely within an 8-h window starting at 10 AM; fasting is then enforced for the subsequent 16 h, beginning at 6 PM. During the trial, there are no restrictions on the types or amounts of food consumed. Participants are encouraged to drink plenty of water and are not restricted in consuming zero-calorie beverages during the fasting period. The trial recruited 40 consenting participants (including eleven excluded for not meeting inclusion criteria, six eligible participants who refused to participate, and six who dropped out), all with a BMI between 30 and 45 kg/m^2^ and ages ranging from 25 to 65 years. The control group used data from a weight loss trial conducted by the organizers in 2011, with the main outcome measured as the percentage of weight loss among participants. In practice, one of the major reasons people struggle to lose weight successfully is low compliance rate with actual weight loss plans. From the literature, studies such as those by Graeme et al. [17] indicate that higher compliance is associated with greater weight loss. Therefore, a clear quantitative analysis of the impact of compliance in weight loss trials is necessary. In this trial, since the daily outcomes reported by participants may include instances of non-compliance, employing a stratified framework is meaningful. For example, always-compliers and never-compliers respectively correspond to subjects who consistently follow the fasting regimen and those who do not adhere to it at all. By collecting baseline covariatessuch as race, gender, age, BMI, blood pressure, and blood sugar levels from the participants, it is possible to appropriately model compliance rates. When adjusting for efficacy, it is also necessary to predict participants’ compliance status based on different baseline covariates. This approach seeks to aid in the future design of weight loss therapies with the aim of improving success rates, which is crucial given the growing global health issues associated with the rising obesity rates.

### 4.2. Simulation Setting

Based on the motivated example discussed above, we analyze the empirical performance of the proposed design under high-dimensional baseline covariates. We consider a two-arm randomized trial, initially recruiting 30 subjects to initiate our sequential monitoring program, with a cohort size of 5. The maximum sample size is set to n.max=100. For the covariates and true compliance model, we first simulate the true compliance status using logistic regression for both linear and nonlinear scenarios:

(1) In the linear case, we generate 20 covariates, $X=(X_{1},X_{2},\ldots ,X_{20})$, where:
  (4) $$\begin{array}{cc} \; & X_{1},X_{2},\ldots ,X_{10}\overset{i.i.d}{∼}\mathrm{Uniform}\left(0,2\right),\\ \; & X_{11},X_{12},\ldots ,X_{15}\overset{i.i.d}{∼}\mathrm{Bern}\left(0.5\right),\\ \; & {\left(X_{16},X_{17},\ldots ,X_{20}\right)}^{T}∼\mathrm{Normal}\left(0,\Sigma \right). \end{array}$$
  Here, $0={\left(0,0,\ldots ,0\right)}^{T}$ is a five-dimensional zero vector, while Σ is the covariance matrix with $\sigma_{ii}=1$ and $\sigma_{ij}=0.5$ (1≤i≠j≤5). The compliance stratum is generated from a logistic regression model:
  (5) $$P\left(S=c\right)=\frac{\exp(\beta_{0}+\beta_{1}X_{1}+\beta_{2}X_{2}+\beta_{3}X_{3}+\beta_{4}X_{11}+\beta_{5}X_{16})}{1+\exp(\beta_{0}+\beta_{1}X_{1}+\beta_{2}X_{2}+\beta_{3}X_{3}+\beta_{4}X_{11}+\beta_{5}X_{16})}$$
  and the other two strats, “always-takers” and “never-takers”, are generated with equal probability from the remaining samples. For regression coefficients $\beta ={\left(\beta_{0},\beta_{1},\beta_{2},\beta_{3},\beta_{4},\beta_{5}\right)}^{T}$ we chose $\beta ={\left(1,8.5,-8.5,8.5,8.5,8.5\right)}^{T}$, allowing us to achieve a high compliance level of 90%; we choose β=(1.2,8.5,−8,8.5,−8.5,8.5) for a medium compliance level of 75%, and for low compliance of 60% we use $\beta ={\left(-1,8.5,9.5,-8.5,-8.5,-8.5\right)}^{T}$. In other words, only five covariates, $X_{1},X_{2},X_{3},X_{11},X_{16}$, are useful in determining the compliance stratum.
(2) In the nonlinear case, we generate 30 covariates:
  (6) $$\begin{array}{cc} \; & X_{1},X_{2},\ldots ,X_{10}\overset{i.i.d}{∼}\mathrm{Uniform}\left(0,2\right),\\ \; & X_{11},X_{12},\ldots ,X_{20}\overset{i.i.d}{∼}\mathrm{Bern}\left(0.5\right),\\ \; & {\left(X_{21},X_{22},\ldots ,X_{30}\right)}^{T}∼N\left(0,\Sigma \right), \end{array}$$
  where $0={\left(0,0,\ldots ,0\right)}^{T}$ is a ten-dimensional zero vector while Σ is the covariance matrix with $\sigma_{ii}=1$ and $\sigma_{ij}=0.5$ (1≤i≠j≤10). The compliance stratum is generated from a logistic regression model
  (7) $$P\left(S=c\right)=\frac{\exp\{\beta_{0}+\beta_{1}X_{1}+\beta_{2}\sqrt{X_{2}}+\beta_{3}X_{11}+\beta_{4}X_{11}X_{21}+\beta_{5}\sin\left(X_{21}\right)+\beta_{6}X_{22}^{2}\}}{1+\exp\{\beta_{0}+\beta_{1}X_{1}+\beta_{2}\sqrt{X_{2}}+\beta_{3}X_{11}+\beta_{4}X_{11}X_{21}+\beta_{5}\sin\left(X_{21}\right)+\beta_{6}X_{22}^{2}\}},$$
  and “’always-takers” and “never-takers” are generated with equal probability from the remaining samples. For the regression coefficients $\beta ={\left(\beta_{0},\beta_{1},\beta_{2},\beta_{3},\beta_{4},\beta_{5},\beta_{6}\right)}^{T}$, we choose $\beta ={\left(-1.5,8,9,-10,-10,9,8.5\right)}^{T}$ to achieve a high compliance level of 90%, β=(−5,7,8,−10,−10,−11,7) for a medium compliance level of 75%, and $\beta ={\left(-1,7,7,-8,-9.5,10,-8\right)}^{T}$ for a low compliance level of 60%. Still, only five covariates $X_{1},X_{2},X_{11},X_{21},X_{22}$ are useful in determining the compliance stratum.

**Figure 3:**
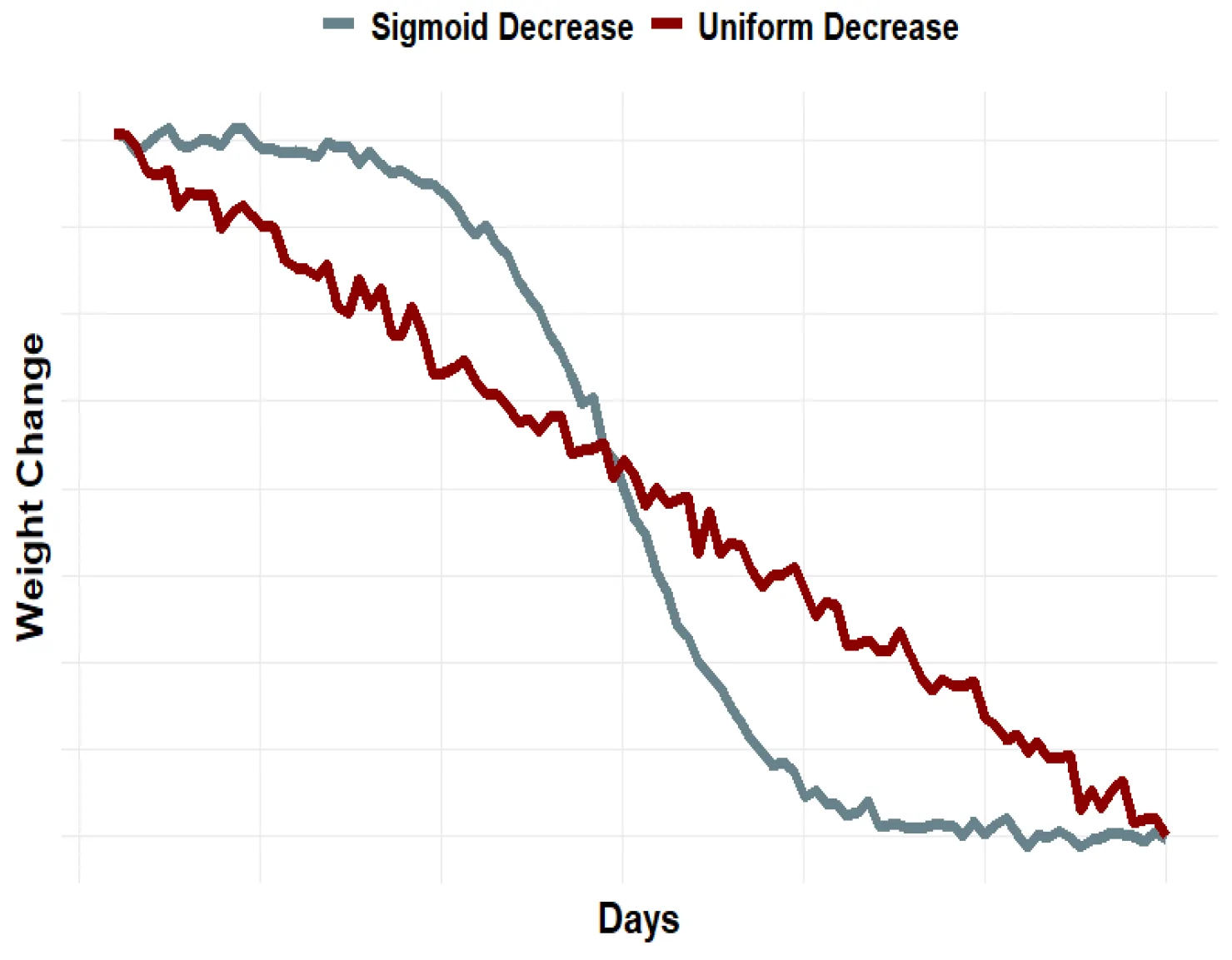
Two common outcome patterns for weight loss: a uniform decline pattern and a sigmoid curve-like decline pattern.

Since our method primarily targets continuous outcome variables, it is essential to consider the actual impact of weight change trends in patients on the CACE. To simulate weight changes in subjects, we adopt two of the most common settings in Figure 3: a weight change pattern that exhibits a uniform decreasing trend (hereafter abbreviated as “Uniform”) and a weight change pattern resembling a sigmoid curve (hereafter abbreviated as “Sigmoid”), where the weight loss effect is primarily evident in the middle to later stages. Based on these two settings, we also simulate the obtained results by directly using mean estimates for comparison. The trial stops when the posterior probability of the CACE magnitude being no greater than L=3 reaches C=0.9, indicating insufficient treatment benefit according to the prespecified futility criterion. Each trial uses 1000 MCMC iterations to fit the model, with the first 400 iterations designated as burn-in. All compliance predictions are implemented using the R package randomForest.

We compare three strategies for handling compliance. The strategy denoted “Our” uses the random forest working classification of the principal strata; “ITT” (Intention-To-Treat) analyzes subjects according to randomized assignment and ignores treatment receipt, thereby estimating the effect of assignment rather than the distributional effect among compliers; and “Gold” uses the true simulated principal strata, which is an oracle benchmark that is unavailable in practice. Within each strategy, we also compare the Wasserstein summary with a mean-only summary; this factorial comparison separates the effect of compliance adjustment from the effect of retaining distributional information. Because the horizontal axis represents the categorical strategies (Our, ITT, and Gold), the revised figures display unconnected points directly above these labels. The Wasserstein and mean summaries are distinguished by high-contrast colors and different point shapes rather than by connecting lines.

**Figure 4:**
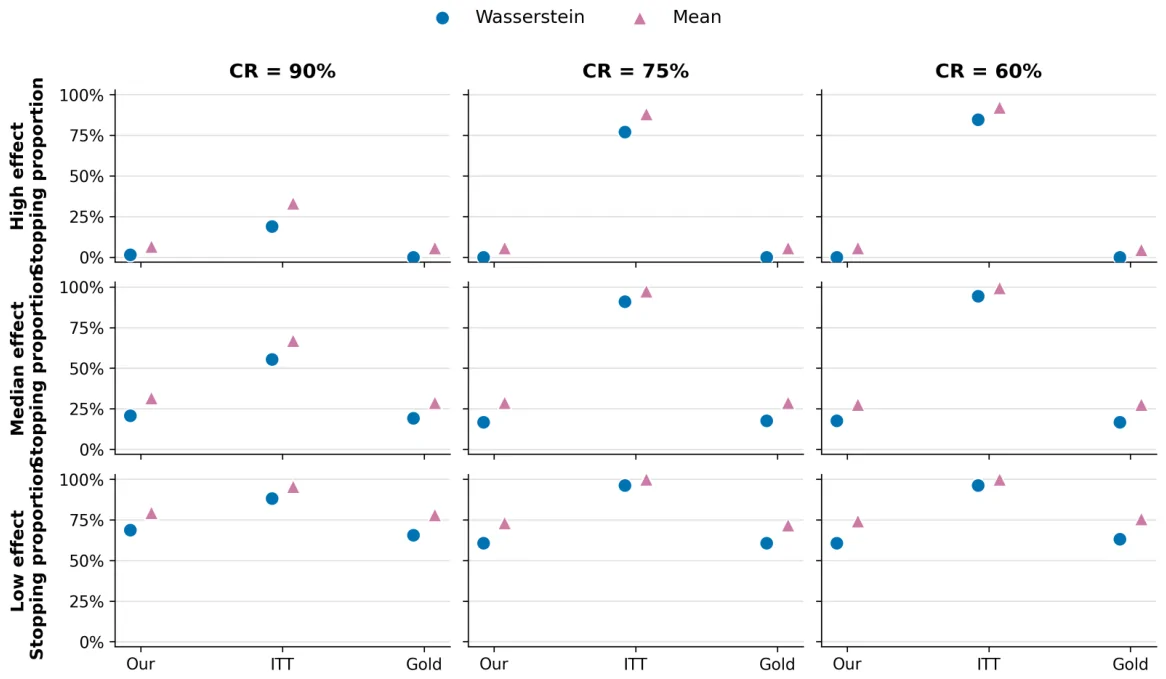
Early stopping proportions under the linear-uniform setting. Points compare Our, ITT, and Gold across the prespecified compliance rates and effect levels for the Wasserstein and mean summaries. The principal comparison is how closely Our follows the oracle (Gold) as compliance decreases.

**Figure 5:**
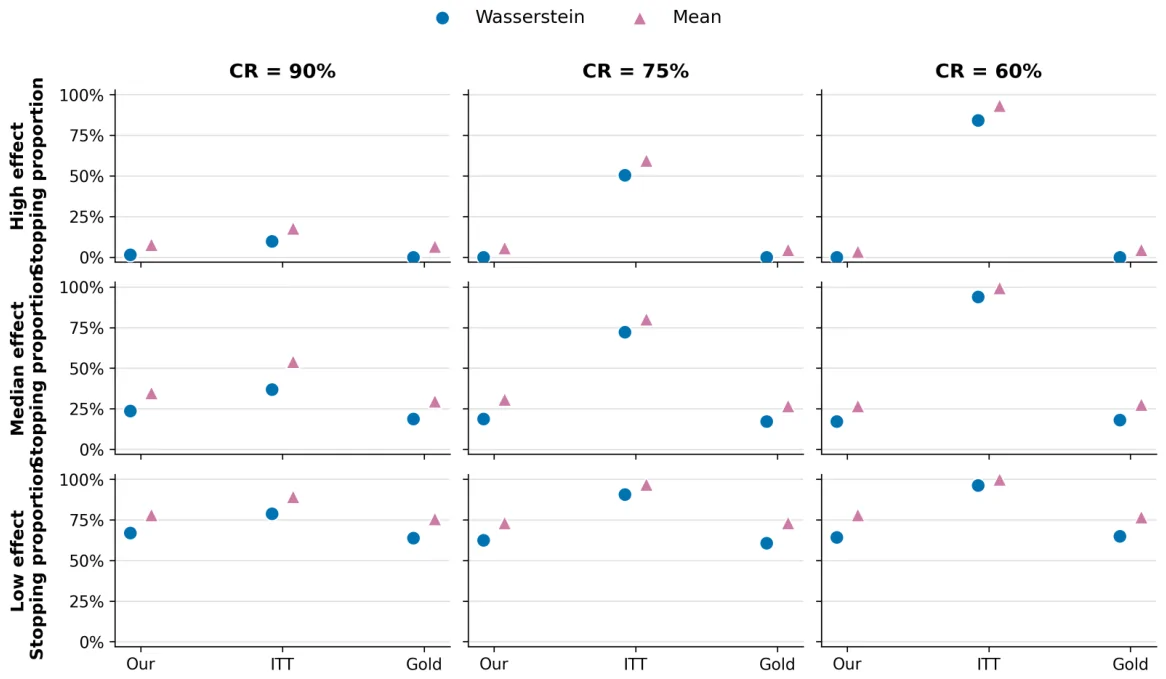
Early stopping proportions under the nonlinear uniform setting. Points compare Our, ITT, and Gold across compliance rates and effect levels for the Wasserstein and mean summaries; divergence of ITT from Gold increases as noncompliance increases.

**Figure 6:**
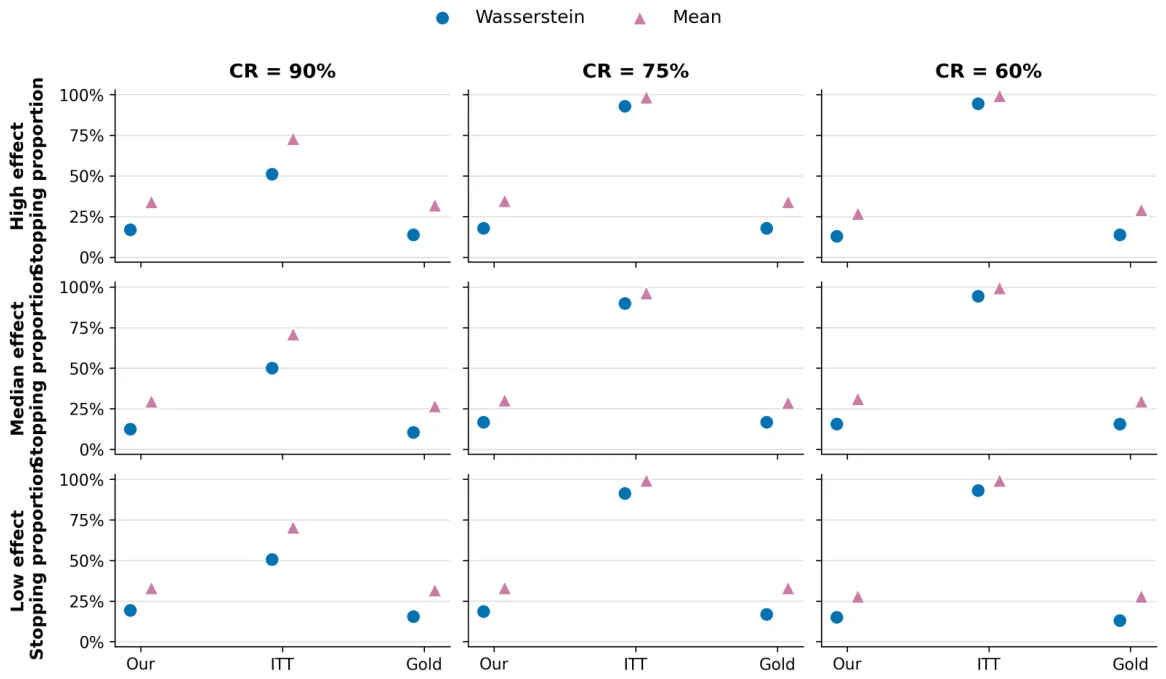
Early stopping proportions under the linear sigmoid setting. Points compare Our, ITT, and Gold (the oracle) across compliance rates and effect levels for the Wasserstein and mean summaries. The Wasserstein summary retains changes concentrated in the middle and later parts of the trajectory.

**Figure 7:**
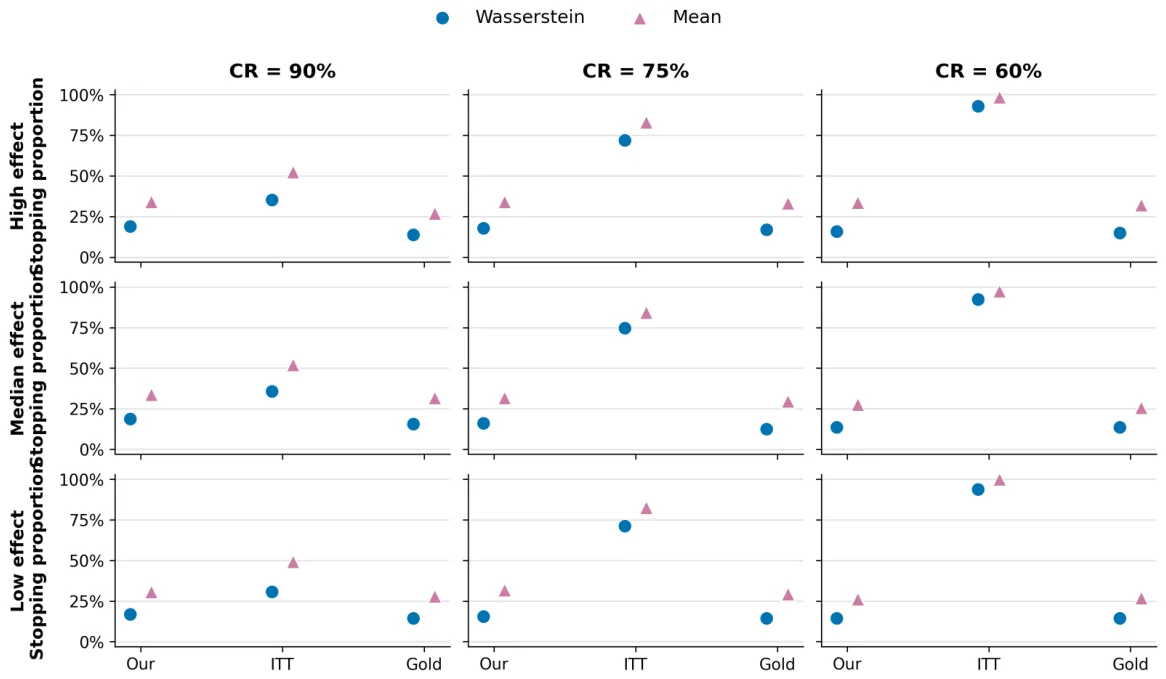
Early stopping proportions under the nonlinear sigmoid setting. Points compare Our, ITT, and Gold across compliance rates and effect levels for the Wasserstein and mean summaries.

Table 1 shows three main patterns: first, as the compliance rate decreases from 90% to 60%, the ITT average sample size departs increasingly from Gold, since ITT does not adjust for treatment received; second, Our remains close to Gold under both the linear and nonlinear compliance mechanisms, indicating that the working classification recovers much of the oracle stopping behavior in the examined scenarios; third, the gap between the Wasserstein and mean summaries is most pronounced for the sigmoid trajectory, where treatment differences are concentrated in the middle and later observation times and so can be attenuated by averaging. These comparisons should be interpreted as operating characteristics under the simulated models. Figure 4, Figure 5, Figure 6 and Figure 7 show the early stopping proportions under the four basic settings in Table 1. Across the examined settings, Our tracks Gold more closely than ITT, and the difference becomes more visible at lower compliance rates.

## 5. Sensitivity Analysis

### 5.1. Results Under Compliance Model Misspecification

We examine sensitivity to compliance model misspecification through a scenario in which an influential covariate is omitted from the fitted model in the presence of high-dimensional baseline covariates. The analysis covers both weight change trajectories and the following two compliance model settings:

(1) In the linear case, we generate 20 covariates $X=(X_{1},X_{2},\ldots ,X_{20})$ in the same way as in (4). The true compliance model is the same as in (5), that is, a logistic regression model with $X_{1},X_{2},X_{3},X_{11}$, and $X_{16}$. We then remove the covariate $X_{1}$ when fitting the compliance model.
(2) In the nonlinear case, we generate a total of 30 covariates as in (6) and the true compliance stratum as in (7), that is, a logistic regression model with $X_{1},X_{2},X_{11},X_{21}$, and $X_{22}$. When fitting the compliance model, we then intentionally ignore the covariate $X_{1}$.

**Figure 8:**
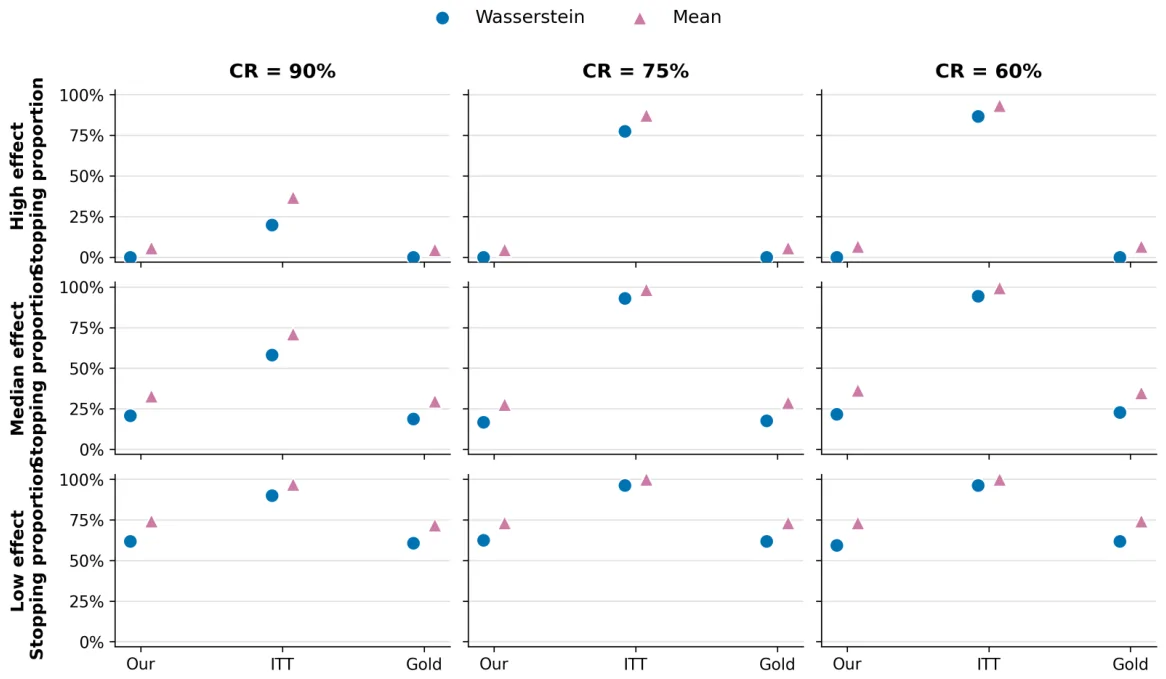
Early stopping proportions under linear uniform compliance model misspecification, with $X_{1}$ omitted. Points compare Our, ITT, and Gold across compliance rates and effect levels for the Wasserstein and mean summaries.

**Figure 9:**
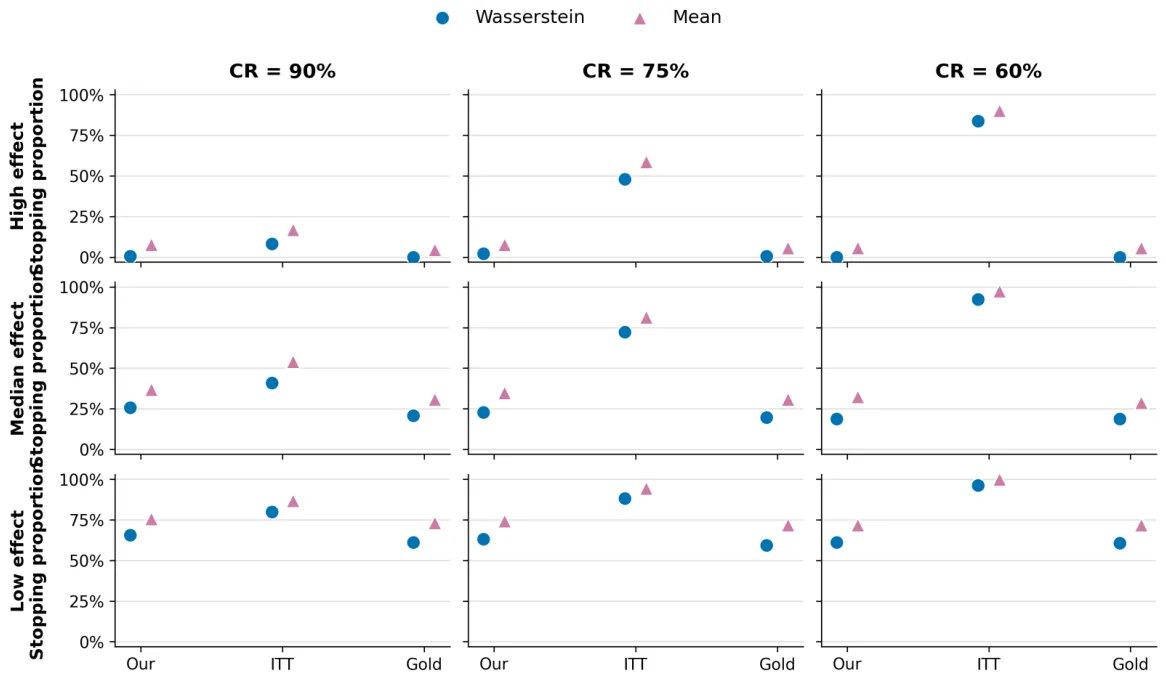
Early stopping proportions under nonlinear uniform compliance model misspecification, with $X_{1}$ omitted. Points compare Our, ITT, and Gold across compliance rates and effect levels for the Wasserstein and mean summaries.

**Figure 10:**
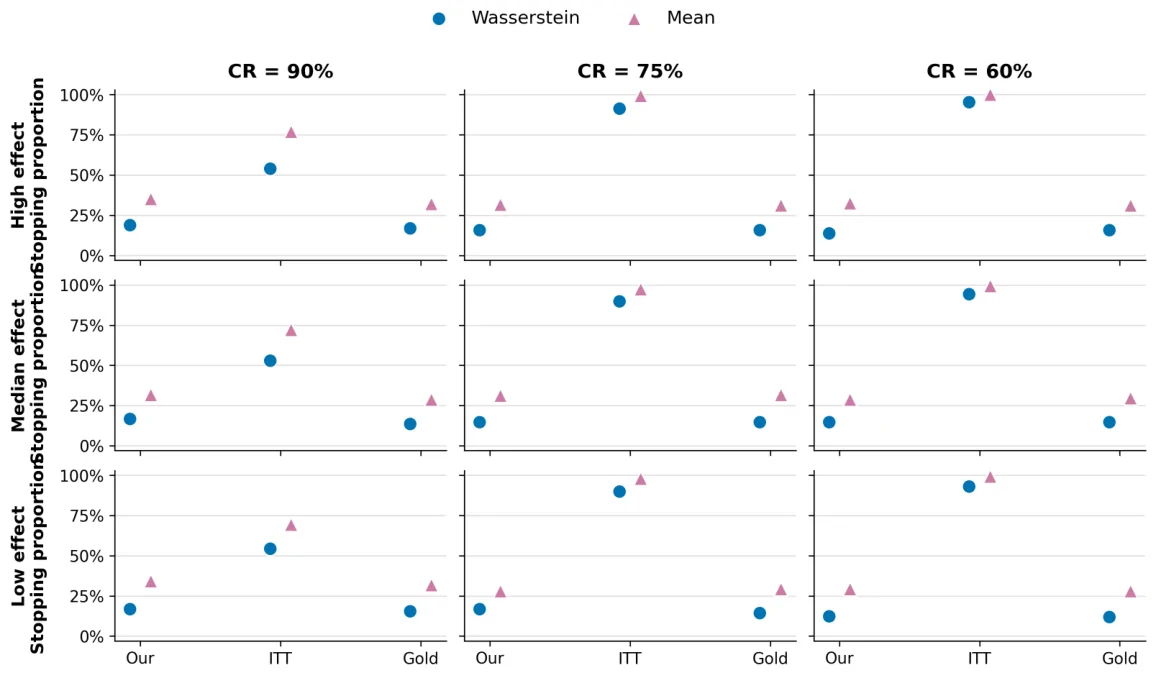
Early stopping proportions under linear sigmoid compliance model misspecification, with $X_{1}$ omitted. Points compare Our, ITT, and Gold across compliance rates and effect levels for the Wasserstein and mean summaries.

**Figure 11:**
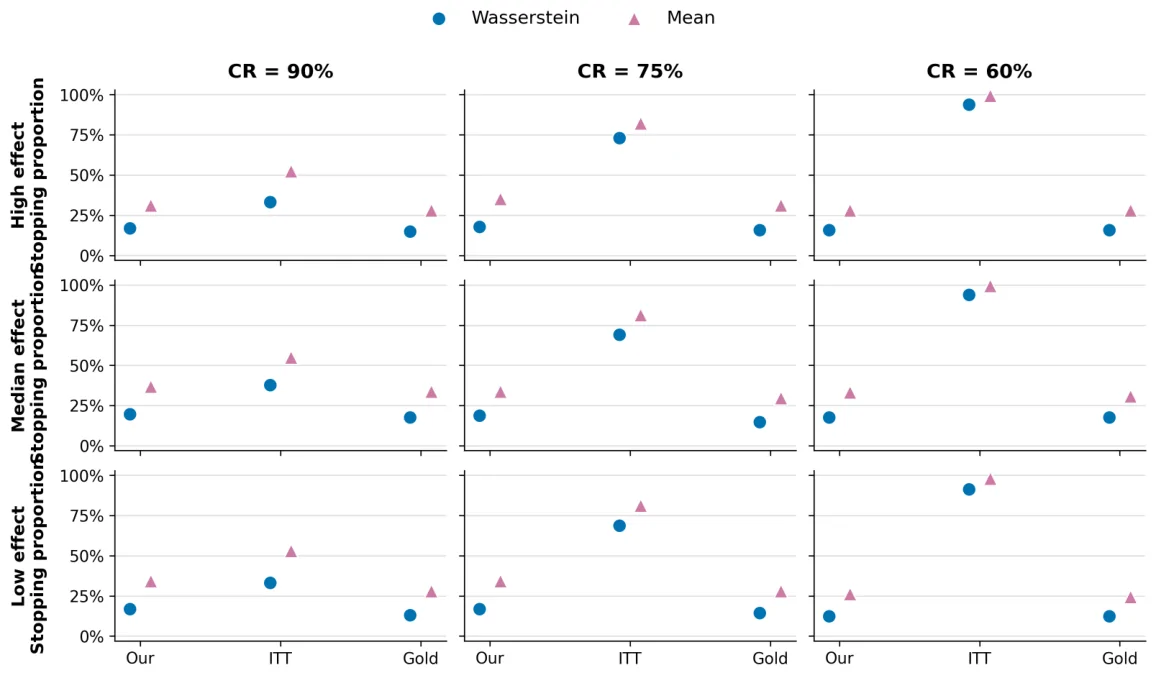
Early stopping proportions under nonlinear sigmoid compliance model misspecification, with $X_{1}$ omitted. Points compare Our, ITT, and Gold across compliance rates and effect levels for the Wasserstein and mean summaries.

Table 2 and Figure 8, Figure 9, Figure 10 and Figure 11 show the average sample size and stopping proportions when the influential covariate $X_{1}$ is omitted from the fitted compliance model. Under both trajectory patterns, Our remains close to Gold under the examined settings. This analysis specifically addresses robustness to a single prespecified omission.

### 5.2. Comparative Analysis

Table 3 summarizes the differences between the Wasserstein and mean-only analyses. Under the Uniform trajectory, the average differences are approximately six subjects in sample size and nine percentage points in early-stopping proportion. Under the Sigmoid trajectory, the corresponding differences are approximately ten subjects and fifteen percentage points. These contrasts are similar between the basic and misspecified compliance-model settings, with changes of less than one subject and approximately one percentage point across corresponding settings. Within the scenarios examined, the results suggest that the choice of distributional summary has a greater influence for the Sigmoid trajectory, in which treatment differences are concentrated in the middle and later observation times.

## 6. Conclusions

In this paper, we have proposed a Bayesian sequential monitoring design for distribution-valued longitudinal outcomes in the presence of noncompliance. The design combines a principal stratification estimand, a working classifier for latent compliance strata, a conjugate Bayesian outcome model, and a Wasserstein effect magnitude used in a posterior futility rule. In the examined simulations, the proposed analysis remains close to the oracle analysis (“Gold”) as compliance decreases, whereas ITT increasingly departs from Gold. The Wasserstein summary is especially useful for trajectory patterns in which clinically relevant differences are attenuated by a mean-only summary. Because ITT and the proposed principal stratification analysis (“Our”) target different estimands, these simulation contrasts should be interpreted as differences in operating characteristics under the investigated scenarios.

Relative to common functional data approaches such as functional principal component analysis, functional regression, and longitudinal mixed models, the proposed method directly targets a distributional causal contrast and embeds it in a stopping design under noncompliance. These features come with limitations: the causal interpretation depends on SUTVA/exclusion and monotonicity, while the numerical analysis depends on the working Gaussian covariance model and the quality of the principal stratum classification. Section 5 examines the effects of omitting one influential compliance predictor; however, this sensitivity analysis does not cover the full range of possible model misspecifications or prior choices. Moreover, because the posterior outcome analysis is conditioned on the predicted principal stratum labels, classification uncertainty is not fully propagated into the posterior distribution of the CACE magnitude.

The computational cost is driven by refitting the random forest and running the Gibbs sampler at each interim look. It grows with the number of interim analyses, retained posterior draws, outcome dimension, and fitted arm/stratum combinations. With a cohort size of five and $n_{\max}=100$, the method is intended for scheduled trial planning and interim analyses rather than real-time bedside computation. More scalable posterior approximations and systematic prior sensitivity analyses are natural extensions.

Application to a real trial would require randomized assignment, recorded treatment receipt, baseline compliance predictors, and longitudinal outcomes observed on a clinically meaningful common scale. The construction of each subject’s outcome distribution, prior hyperparameters, futility threshold *L*, and posterior boundary *C* should be prespecified. The present study evaluates operating characteristics through simulations motivated by a fasting trial; prospective validation or analysis of an appropriate individual-level randomized dataset remains important future work. Other directions include missing longitudinal observations, irregular observation times, additional functional endpoints, and methods that relax the identifying assumptions.

## Figures and Tables

**Table 1:** Average sample size at stopping in the basic simulation settings. “Our” uses the random forest working classification, “ITT” ignores treatment receipt and analyzes randomized assignment, and “Gold” uses the true simulated principal strata. Results are shown for Wasserstein and mean summaries under linear and nonlinear compliance mechanisms, uniform and sigmoid trajectories, and compliance rates (CR) of 90%, 75%, and 60%.

				Wasserstein Samples			Mean Samples		
Change Trend	Model Type	CR	Effect	Our	ITT	Gold	Our	ITT	Gold
Uniform	Linear	90%	Low	54.0	39.3	56.1	46.4	35.1	47.4
			Median	86.8	66.0	87.6	79.5	57.7	81.1
			High	97.9	89.2	98.6	95.4	81.3	96.4
		75%	Low	60.2	31.2	58.9	51.7	30.6	52.0
			Median	89.6	40.2	88.9	82.1	35.6	81.5
			High	98.6	56.3	98.7	96.8	46.2	96.4
		60%	Low	60.0	30.6	58.0	51.0	30.3	49.8
			Median	89.5	36.3	89.2	82.4	33.1	81.7
			High	99.2	49.0	99.4	97.0	42.3	97.1
	Non-linear	90%	Low	54.9	46.3	56.9	46.3	39.1	48.3
			Median	85.2	76.3	87.9	77.5	65.6	80.7
			High	98.5	93.7	98.9	95.3	89.5	96.2
		75%	Low	57.8	36.0	58.3	50.2	33.9	50.0
			Median	88.8	53.4	88.9	80.7	47.5	82.7
			High	99.0	69.0	99.1	96.5	62.7	97.3
		60%	Low	57.8	31.0	55.8	48.6	30.4	47.6
			Median	89.8	34.8	88.4	82.6	32.5	82.1
			High	99.1	48.8	99.0	97.6	41.2	97.4
Sigmoid	Linear	90%	Low	87.7	68.5	89.9	77.8	55.9	79.3
			Median	92.0	69.9	93.3	81.4	56.2	82.8
			High	89.5	68.6	91.2	77.7	55.1	79.5
		75%	Low	89.0	39.9	88.7	78.6	34.3	78.0
			Median	89.9	41.3	89.9	80.8	35.6	81.0
			High	88.4	37.6	88.2	78.1	34.2	77.4
		60%	Low	91.2	35.7	91.4	82.0	32.1	81.6
			Median	90.9	36.4	90.0	80.2	33.0	80.3
			High	91.8	36.1	91.2	83.1	32.9	81.4
	Non-linear	90%	Low	89.2	81.1	91	79.9	69.0	82.0
			Median	88.2	77.7	90.1	77.9	65.8	79.4
			High	88.0	78.6	90.9	77.3	66.1	81.8
		75%	Low	90.2	54.7	90.8	79.5	45.5	80.6
			Median	90.8	52.2	91.9	79.9	44.1	81.5
			High	88.8	54.0	88.8	78.3	46.5	78.8
		60%	Low	90.8	38.1	90.7	83.5	33.0	82.6
			Median	91.4	37.6	91.1	83.0	33.9	82.8
			High	90.7	37.2	90.3	79.9	33.7	79.3

**Table 2:** Average sample size at stopping when the fitted compliance model omits the influential covariate $X_{1}$. “Our” denotes the proposed analysis under this misspecification, “ITT” ignores treatment receipt, and “Gold” uses the true simulated strata. Results cover both trajectory patterns, both compliance mechanisms, and compliance rates (CR) of 90%, 75%, and 60%.

				Wasserstein Samples			Mean Samples		
Change Trend	Model Type	CR	Effect	Our	ITT	Gold	Our	ITT	Gold
Uniform	Linear	90%	Low	58.0	39.0	59.0	49.5	35.4	51.5
			Median	87.1	65.2	88.1	78.4	55.8	80.4
			High	99.2	88.9	99.4	96.8	79.4	97.4
		75%	Low	58.3	31.1	57.2	50.8	30.4	49.8
			Median	89.5	39.9	89.5	82.6	35.6	82.0
			High	98.4	54.9	98.6	97.0	47.5	96.7
		60%	Low	59.8	30.6	58.3	51.5	30.2	50.0
			Median	86.8	35.4	85.4	77.8	33.1	77.1
			High	99.1	47.1	98.8	96.2	40.4	96.2
	Non-linear	90%	Low	55.1	45.8	58.8	48.6	41.3	50.9
			Median	83.1	74.6	86.5	75.8	65.6	79.6
			High	98.7	94.7	99.2	95.4	89.7	96.8
		75%	Low	59.2	36.9	60.2	50.8	35.0	51.3
			Median	87.1	52.7	87.7	78.3	46.5	79.9
			High	98.0	71.5	98.1	95.8	63.8	96.2
		60%	Low	60.1	30.8	59.1	53.0	30.3	51.5
			Median	89.2	35.5	88.2	79.6	33.4	80.9
			High	99.4	49.1	99.2	96.9	43.0	96.7
Sigmoid	Linear	90%	Low	89.2	66.9	89.9	77.5	55.8	79.2
			Median	89.1	68.6	91.4	79.5	55.1	81.7
			High	88.0	66.2	89.7	77.1	51.9	78.9
		75%	Low	89.8	40.3	90.5	81.7	35.7	81.2
			Median	91.2	41.6	90.9	80.3	35.6	79.3
			High	90.8	41.8	90.2	80.0	35.9	79.5
		60%	Low	92.6	37.3	92.3	81.6	33.5	81.4
			Median	91.0	36.5	90.7	82.3	32.8	81.4
			High	91.8	35.8	90.2	79.9	32.3	79.7
	Non-linear	90%	Low	89.2	81.1	91	79.9	69.0	82.0
			Median	87.5	77.0	89.0	76.5	64.6	78.6
			High	88.9	79.4	90.5	79.9	67.4	81.8
		75%	Low	90.2	56.4	90.7	79.5	46.8	81.3
			Median	89.0	55.2	90.4	79.1	46.7	80.3
			High	89.0	53.2	89.9	78.5	45.8	79.3
		60%	Low	92.6	38.4	91.8	84.4	34.0	83.9
			Median	89.0	36.6	88.7	78.9	33.1	79.7
			High	90.0	36.7	89.9	82.3	33.5	81.7

**Table 3:** Differences in average sample size and stopping rates between our method and mean estimates under two weight change trends, at different compliance rates and average differences. Results are calculated as Wasserstein estimate minus the mean estimate.

			Difference in Sample Size				Difference in Early Termination			
Change Trend	Model Type	Fitting Method	90%	75%	60%	Average	90%	75%	60%	Average
Uniform	Linear	Basic	5.8	5.9	6.1	5.9	8.0%	9.3%	9.1%	8.8%
		Misspecification	6.5	5.3	6.7	6.2	9.7%	7.9%	10.8%	9.5%
	Nonlinear	Basic	6.5	6.1	6.0	6.2	9.3%	9.0%	8.6%	9.0%
		Misspecification	5.7	6.5	6.3	6.2	8.2%	9.0%	9.1%	8.8%
Sigmoid	Linear	Basic	10.8	9.9	9.5	10.1	15.9%	15.0%	14.6%	15.2%
		Misspecification	10.7	9.9	10.4	10.3	16.1%	14.3%	16.2%	15.5%
	Nonlinear	Basic	10.1	10.7	8.8	9.9	15.1%	16.1%	13.9%	15.0%
		Misspecification	10.6	10.4	8.7	9.9	16.1%	16.4%	13.4%	15.3%

## Data Availability

No new data were created or analyzed in this study. Data sharing is not applicable to this article.
